# Incidence Trends and Geographic Variations of Corpus Uteri and Cervical Cancer in Taiwan, 1995–2022: A Population-Based Study

**DOI:** 10.3390/cancers18050881

**Published:** 2026-03-09

**Authors:** Yu Chang, Nari Kay, Liang-Chun Chiu, Chung-I Huang, Hung-Ju Li, Shyh-An Yeh, Yu-Chieh Su

**Affiliations:** 1 Department of Obstetrics and Gynecology, Kaohsiung Show Chwan Memorial Hospital, Kaohsiung 82151, Taiwan; 8lg001@ksch.org.tw (Y.C.); 8lg006@ksch.org.tw (N.K.); 2 Department of Nursing, Meiho University, Ping Tung 91202, Taiwan; 3 Department of Obstetrics and Gynecology, E-Da Dachang Hospital, I-Shou University, Kaohsiung 80794, Taiwan; 4 Department of Radiation Oncology, E-Da Cancer Hospital, I-Shou University, Kaohsiung 82445, Taiwan; ed108856@edah.org.tw; 5 Department of Medical Imaging and Radiological Sciences, College of Medical Science and Technology, I-Shou University, Kaohsiung 82445, Taiwan; 6 Division of Hematology-Oncology, Department of Internal Medicine, E-Da Hospital, I-Shou University, Kaohsiung 82445, Taiwan; 7 Department of Radiation Oncology, E-Da Hospital, I-Shou University, Kaohsiung 82445, Taiwan; 8 Graduate Institute of Medicine, College of Medicine, I-Shou University, Kaohsiung 82445, Taiwan; 9 School of Medicine, College of Medicine, I-Shou University, Kaohsiung 82445, Taiwan

**Keywords:** age–period–cohort analysis, cervical cancer, uterine corpus cancer, incidence trends, Taiwan

## Abstract

Background/Objectives: To examine long-term incidence trends of cervical cancer and corpus uteri cancer in Taiwan from 1995 to 2022, with emphasis on age–period–cohort patterns and regional variation. Methods: Data from the Taiwan cancer registry were analyzed. Age-standardized incidence rates (ASRs) were calculated using the 1976 World Standard Population. Temporal trends were evaluated using Joinpoint regression to estimate annual percent changes (APCs) and average annual percent changes (AAPCs). Age–period–cohort modeling was applied to assess net drift, cohort effects, and period effects. Subgroup analyses were conducted by geographic region and urbanization level. Results: Cervical cancer incidence declined markedly, with ASRs decreasing from 20.06 to 6.78 per 100,000 women between 1995 and 2022 (AAPC = −4.43%, 95% CI: −5.39 to −3.45). In contrast, corpus uteri cancer incidence increased substantially, with ASRs rising from 2.91 to 17.42 per 100,000 women (AAPC = 6.32%, 95% CI: 5.86–6.78). Age–period–cohort analysis revealed a negative net drift for cervical cancer (−5.0% per year) and a positive net drift for corpus uteri cancer (6.1% per year). Cohort effects indicated decreasing cervical cancer risk among women born after 1960, whereas corpus uteri cancer risk increased in successive younger cohorts. Period effects showed pronounced declines in cervical cancer incidence after 2000, patterns that are compatible with the implementation of organized screening, while corpus uteri cancer continued to rise. Conclusions: Cervical cancer incidence in Taiwan has declined substantially over the past three decades, a pattern that is compatible with the long-term impact of organized screening programs. In contrast, the increasing burden of corpus uteri cancer may be associated with generational shifts in metabolic and reproductive risk factors.

## Simple Summary

Cervical cancer and cancer of the uterine body show opposite trends in many countries, but long-term population-based evidence from Asia remains limited. Using cancer registry data from Taiwan between 1995 and 2022, we examined how the incidence of these two cancers has changed over time and whether patterns differed by age, birth cohort, geographic region, and urbanization level. We found that cervical cancer incidence has declined substantially over the past three decades, a pattern that is compatible with the long-term impact of organized screening programs, whereas cancer of the uterine body has increased steadily across generations. These contrasting trends underscore the differing public health challenges posed by cervical and uterine body cancers. Our findings provide important population-level evidence to inform future cancer prevention strategies and healthcare planning.

## 1. Introduction

Over the past three decades, the incidence of uterine corpus cancer has increased substantially in many high-income populations worldwide, including Taiwan [1,2,3,4]. Using cancer registry data from Taiwan, Tai et al. reported that the age-standardized incidence rate (ASR) nearly tripled between 1998 and 2017, with the most pronounced increase observed in endometrioid carcinoma [2]. Non-endometrioid carcinoma and uterine sarcoma also exhibited upward incidence trends, although their absolute incidence remained relatively low. Similar increasing patterns across age groups and successive birth cohorts have been reported in other populations, suggesting the influence of generational shifts in reproductive patterns, metabolic health, and lifestyle factors [2,5,6,7].

In contrast, cervical cancer incidence in Taiwan has declined over the past two decades. Chiang et al. demonstrated a substantial reduction in ASR between 2002 and 2012, temporally associated with the implementation of a cytology-based screening program in Taiwan [8]. Persistent infection with high-risk human papillomavirus (HPV) remains the necessary cause of cervical cancer, and organized screening programs have been shown to effectively reduce incidence and mortality through the early detection and treatment of precancerous lesions [9,10,11].

Globally, divergent trends in these two gynecologic malignancies have been observed. Uterine corpus cancer has become the most common gynecologic cancer in many high-income countries, driven by population aging and rising prevalence of obesity and metabolic disorders [12]. In contrast, cervical cancer incidence has declined substantially in regions with established screening programs, yet it remains a major cause of cancer-related mortality in low- and middle-income countries, reflecting persistent global disparities in access to prevention and care [13].

Socioeconomic development and healthcare infrastructure play critical roles in shaping cancer incidence patterns [3,14,15]. Regional variations in screening uptake, healthcare accessibility, reproductive behaviors, and lifestyle factors may contribute to heterogeneous trends across geographic areas and population groups [16,17]. However, in Taiwan, comprehensive long-term analyses integrating age–period–cohort effects with geographic and urbanization variations remain limited, particularly those examining both corpus uteri and cervical cancers within the same population framework.

Therefore, this study aimed to examine incidence trends of uterine corpus cancer and cervical cancer in Taiwan from 1995 to 2022 using population-based registry data. We evaluated temporal patterns by age, birth cohort, geographic region (northern, central, southern, and eastern Taiwan), and urbanization level (metropolitan vs. non-metropolitan areas), with the goal of providing updated epidemiological evidence to inform cancer prevention strategies and health system planning.

## 2. Materials and Methods

### 2.1. Data Sources

We used population-based registry data between 1995 and 2022 from Taiwan. The data were obtained from the Cancer Registry Annual Report published by the Health Promotion Administration, Ministry of Health and Welfare, Taiwan . These annual reports are derived from the Taiwan Cancer Registry Database, a population-based cancer registry system with high-quality indicators. The completeness rate of the registry increased from 91.3% in 2001 to 98.1% in 2020. The proportion of death-certificate-only cases, which indicates incomplete case ascertainment, declined from 8.84% in 1998 to 0.75% in 2022 [18].

### 2.2. Case Definition

According to the 2022 Taiwan Cancer Registry classification, cervical cancer was defined by ICD-O-3 code C53, and corpus uteri cancer by ICD-O-3 code C54. Corpus uteri cancers were further categorized into three histological groups: (1) Endometrial carcinoma, including endometrioid adenocarcinoma (ICD-O-3: 8263/3, 8380/3, 8382/3, 8570/3), serous carcinoma (ICD-O-3: 8441/3, 8461/3), clear cell carcinoma (ICD-O-3: 8310/3), and other specified epithelial carcinomas (ICD-O-3: 8013/3, 8020/3, 8041/3, 8070/3, 8140/3, 8255/3, 8323/3, 8480/3, 8574/3). (2) Uterine sarcoma, including leiomyosarcoma (ICD-O-3: 8890/3, 8891/3, 8896/3, 8897/3), endometrial stromal sarcoma (ICD-O-3: 8805/3, 8930/3, 8931/3), and other sarcomas (ICD-O-3: 8714/3, 8800/3, 8825/3, 8920/3, 8933/3, 9540/3). (3) Others, including carcinoma not otherwise specified (NOS) (ICD-O-3: 8010/3), other specified carcinomas (ICD-O-3: 8144/3, 8249/3, 8803/3, 9111/3), and miscellaneous malignancies (ICD-O-3: 8000/3, 8900/3, 8910/3, 8963/3, 9100/3, 9110/3, 9120/3, 9581/3). To maintain consistency with the registry, cases listed as “non-specified epithelial carcinoma” (ICD-O-3: 8010/3) were classified under the “others” category. For cervical cancer, cases were further classified into three major histological subtypes according to ICD-O-3 morphology codes: (1) Squamous cell carcinoma (ICD-O-3: 8052/3, 8070/3, 8071/3, 8072/3, 8076/3, 8083/3, 8085/3, 8086/3); (2) Adenocarcinoma (ICD-O-3: 8140/3, 8263/3, 8310/3, 8380/3, 8384/3, 8441/3, 8480/3, 8482/3, 8483/3, 8484/3, 8574/3, 9110/3); (3) Others, including adenosquamous carcinoma (ICD-O-3: 8560/3), neuroendocrine carcinoma (ICD-O-3: 8041/3, 8240/3, 8246/3), other specified carcinomas (ICD-O-3: 8020/3, 8032/3, 8045/3, 8098/3), sarcomas (ICD-O-3: 8800/3, 8801/3, 8890/3, 8910/3, 9364/3), malignant melanoma (ICD-O-3: 8720/3), mixed epithelial and sarcoma (ICD-O-3: 8933/3, 8980/3), and carcinoma not otherwise specified (ICD-O-3: 8010/3, 8000/3).

### 2.3. Statistical Analysis

The crude incidence rate and ASR per 100,000 women were calculated. ASRs were age-adjusted to the 1976 World Standard Population. Age-specific incidence rates were calculated for three age categories: 20–39, 40–59, and ≥60 years, and expressed per 100,000 women.

To evaluate temporal trends, annual percent change (APC) and average annual percent change (AAPC) were estimated using Joinpoint regression analysis (version 4.9.1.0; NCI Statistical Methodology and Applications Branch, Bethesda, MD, USA) [19]. An independent two-sided *t*-test was used to determine whether APCs and AAPCs differed significantly from zero, with *p* < 0.05 considered statistically significant.

For regional analysis, Taiwan was divided into four geographic regions: northern, central, southern, and eastern Taiwan. Urbanization level was categorized as metropolitan (the six special municipalities: Taipei, New Taipei, Taoyuan, Taichung, Tainan, and Kaohsiung) or non-metropolitan (all other areas). This classification was based on administrative units as reported in the Taiwan Cancer Registry and does not reflect finer township- or district-level urbanization characteristics.

In addition, an age–period–cohort analysis was performed using the NCI APC Web Tool (https://analysistools.cancer.gov/apc/,accessed on 26 August 2025) [20]. This model used 5-year age groups (20–24, 25–29, …, ≥85) and 5-year calendar periods to estimate net drift (overall annual percent change), local drifts (age-specific annual percent changes), cohort effects, and period effects, following the methodology described by Rosenberg et al. [20].

## 3. Results

### 3.1. Corpus Uteri Cancer

**Figure 1:**
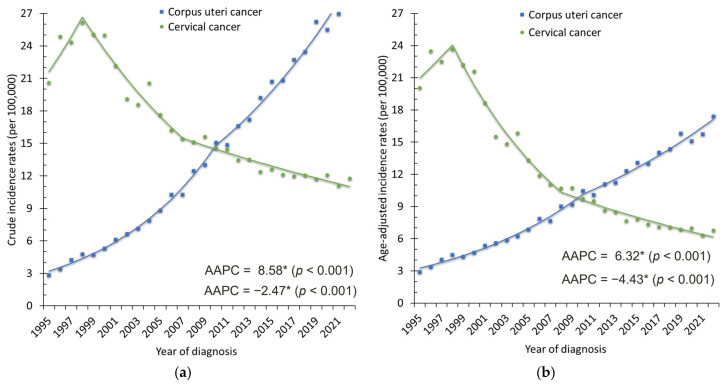
(**a**) Crude incidence rates; (**b**) age-standardized incidence rates (ASRs) of corpus uteri and cervical cancers in Taiwan, 1995–2022. Dots represent the observed annual incidence rates, and the solid lines represent the trends estimated using Joinpoint regression. * indicates that the AAPC is statistically significant.

**Figure 2:**
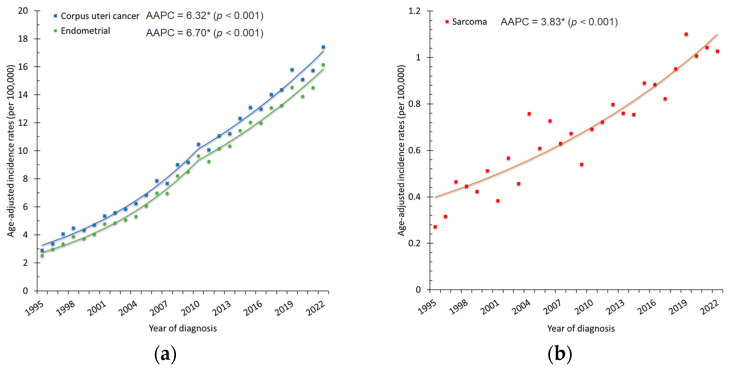
Age-standardized incidence rates (ASRs) of corpus uteri cancer by histologic type: (**a**) endometrial carcinoma and overall corpus uteri cancer; (**b**) uterine sarcoma. Dots represent the observed annual incidence rates, and the solid lines represent the trends estimated using Joinpoint regression. * indicates that the AAPC is statistically significant.

**Figure 3:**
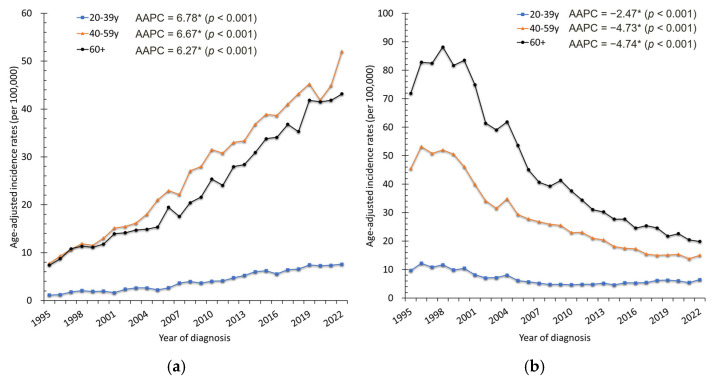
Crude age-specific incidence rate trends of (**a**) corpus uteri cancer and (**b**) cervical cancer, Taiwan, 1995–2022. * indicates that the AAPC is statistically significant.

From 1995 to 2022, a total of 44,933 women in Taiwan were diagnosed with corpus uteri cancer, of whom 41,083 (91.4%) had endometrial carcinoma, 3130 (7.0%) had uterine sarcoma, and the remaining 720 were classified as other histologic types. The crude incidence of corpus uteri cancer increased markedly, rising from 295 cases (2.85 per 100,000 women) to 3541 cases (30.10 per 100,000 women) (Figure 1a). The ASR rose from 2.91 to 17.42 per 100,000 women, with an AAPC of 6.32% (95% CI: 5.86–6.78%, *p* < 0.001) (Figure 1b). Endometrial carcinoma was the predominant histology, accounting for more than 90% of cases, with its ASR increasing from 2.53 to 16.16 per 100,000 women (AAPC = 6.70%, 95% CI: 6.22–7.19%, *p* < 0.001). Uterine sarcoma also showed a smaller but significant rise (AAPC = 3.83%, 95% CI: 3.19–4.47%, *p* < 0.001) (Figure 2). Age-specific analysis revealed significant increases across all groups: AAPCs were 6.78% (95% CI: 5.81–7.76%) for ages 20–39, 6.67% (95% CI: 6.11–7.23%) for ages 40–59, and 6.27% (95% CI: 5.73–6.82%) for those aged ≥60 years (all *p* < 0.001). These trends were consistent with the overall upward trajectory of corpus uteri cancer incidence (Figure 3a).

**Figure 4:**
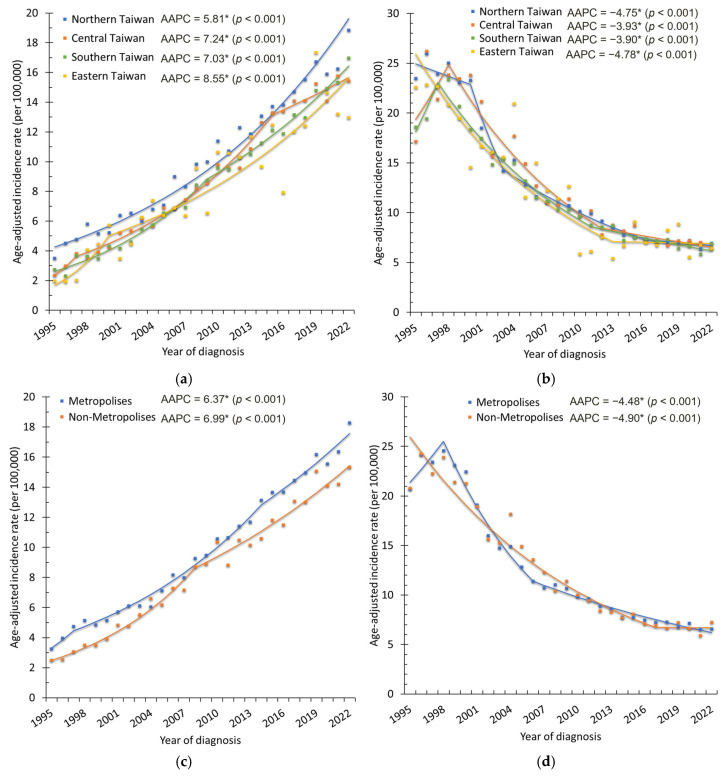
Age-standardized incidence rate (ASR) trends of corpus uteri cancer and cervical cancer by (**a**) geographic region, corpus uteri cancer; (**b**) geographic region, cervical cancer; (**c**) urbanization level, corpus uteri cancer; and (**d**) urbanization level, cervical cancer, 1995–2022. Dots represent the observed annual incidence rates, and the solid lines represent the trends estimated using Joinpoint regression. * indicates that the AAPC is statistically significant.

By geographic region (Figure 4a and Table 1), ASRs increased significantly in all four regions of Taiwan (all *p* < 0.001). The steepest increase occurred in Central Taiwan (APC = 23.66% during 1995–1997, 95% CI: 4.11–46.88%, *p* = 0.018), followed by a sustained increase of 7.41% per year, with a subsequent deceleration in later years. Southern Taiwan also showed a marked increase (APC = 9.06% during 1995–2009), while eastern Taiwan had the steepest initial growth (APC = 24.09% during 1995–2000), before moderating to a more gradual increase. By urbanization level (Figure 4c, Table 2), both metropolitan and non-metropolitan areas experienced significant increases, with slightly faster growth in non-metropolitan areas (AAPC = 6.99% vs. 6.37%, both *p* < 0.001).

**Figure 5:**
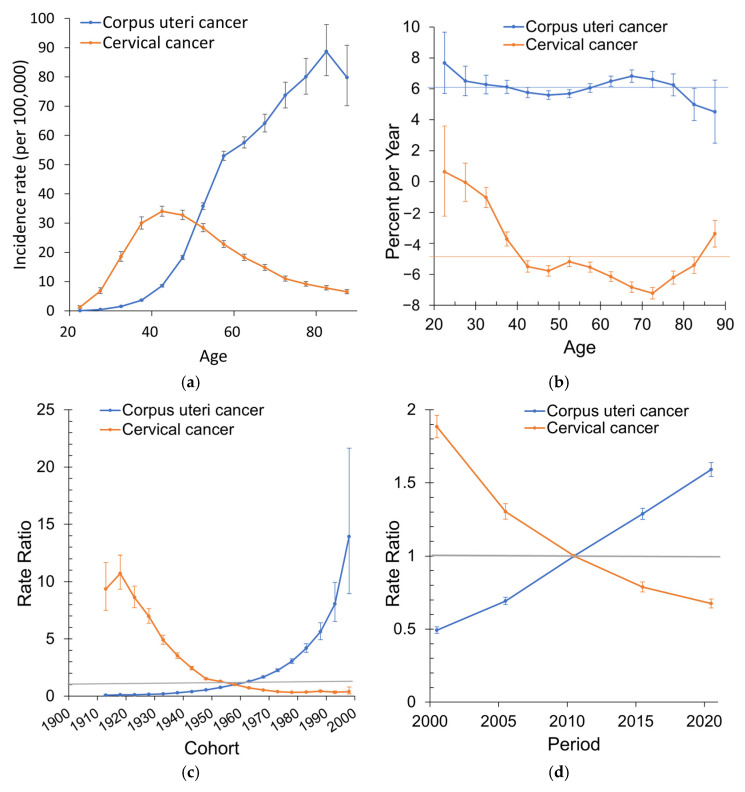
Age–period–cohort analysis of corpus uteri cancer and cervical cancer in Taiwan, 1998–2022: (**a**) longitudinal age curves (incidence rates plotted at the midpoint of each age group, per 100,000 person-years); (**b**) local drifts with net drift (horizontal line indicates overall annual percent change); (**c**) cohort relative risks; (**d**) period relative risks. Rate ratios greater than 1 indicate higher incidence relative to the reference cohort or period, whereas values below 1 indicate lower incidence. The reference cohort and reference period correspond to the central categories specified in the age–period–cohort model. The grey horizontal line indicates a rate ratio of 1, representing the reference level.

Age–period–cohort modeling further demonstrated a significant net drift of +6.1% per year (95% CI: 5.9–6.3%). Local drift estimates indicated consistently positive increases across all age groups, particularly in younger women. The longitudinal age curve showed the incidence rising steadily with age, peaking at 88.7 per 100,000 at the 80–84 year age group (midpoint 82.5 years). Cohort effects revealed a steady increase in relative risk among successive birth cohorts, with women born after 1958 experiencing significantly higher risks. Period effects also showed consistent increases, with rate ratios rising from 0.49 in 2000.5 to 1.59 in 2020.5, relative to the reference year 2010.5 (Figure 5).

### 3.2. Cervical Cancer

**Figure 6:**
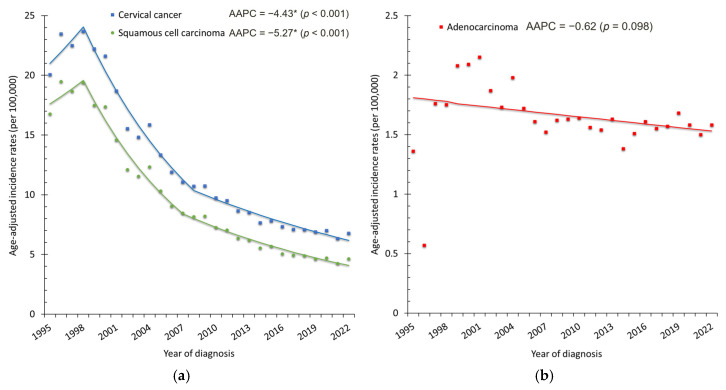
Age-standardized incidence rates (ASRs) of cervical cancer by histologic type: (**a**) squamous cell carcinoma and overall cervical cancer; (**b**) adenocarcinoma. Dots represent the observed annual incidence rates, and the solid lines represent the trends estimated using Joinpoint regression. * indicates that the AAPC is statistically significant.

In contrast, cervical cancer incidence declined substantially from 1995 to 2022. A total of 52,740 women were diagnosed with cervical cancer. Of these, 40,349 (76.5%) were squamous cell carcinoma, 7179 (13.6%) were adenocarcinoma, and the remaining 5212 (9.9%) were classified as other histologic types. Crude cases decreased from 2136 (20.60 per 100,000) to 1384 (11.76 per 100,000) (Figure 1a). The ASR dropped from 20.06 to 6.78 per 100,000, corresponding to an AAPC of −4.43% (95% CI: −5.39 to −3.45%, *p* < 0.001) (Figure 1b). Squamous cell carcinoma was the predominant histology throughout the study period, accounting for more than 75% of cases. Its ASR decreased markedly from 16.77 to 4.64 per 100,000 women (AAPC = −5.27%, 95% CI: −6.30 to −4.23%, *p* < 0.001). In contrast, adenocarcinoma demonstrated a relatively stable trend over time (AAPC = −0.62%, 95% CI: −1.36 to 0.12%, *p* = 0.098) (Figure 6). Age-specific analysis confirmed significant declines across all groups: AAPCs were −2.47% (95% CI: −3.35 to −1.38%) for ages 20–39, −4.73% (95% CI: −6.22 to −3.22%) for ages 40–59, and −4.74% (95% CI: −5.75 to −3.72%) for those aged ≥60 years (all *p* < 0.001). These age-specific patterns mirrored the overall downward trend (Figure 3b).

By geographic region (Figure 4b and Table 1), significant decreases were observed in all four regions (AAPCs ranging from −3.90% to −4.78%, *p* < 0.001). The most pronounced decreases were recorded in northern Taiwan (APC = −14.46% during 2000–2003) and central Taiwan (APC = −7.41% during 1998–2012). Southern Taiwan also showed steady declines (APC = −6.79% during 1997–2011), whereas eastern Taiwan exhibited a plateau after 2013, with no significant change thereafter (APC = −0.30%, *p* = 0.907). By urbanization level (Figure 4d, Table 2), both metropolitan and non-metropolitan areas experienced significant decreases (AAPC = −4.48% and −4.90%, respectively; both *p* < 0.001). In metropolitan areas, the most rapid decrease occurred during 1998–2006 (APC = −9.57%), followed by a slower decline in 2006–2022 (APC = −3.73%). In non-metropolitan areas, the decline remained steep until 2017 (APC = −5.96%), after which rates plateaued with no significant change during 2017–2022 (APC = −0.10%, *p* = 0.972).

Age–period–cohort modeling revealed a significant net drift of −5.0% per year (95% CI: −5.2 to −4.8%). Local drifts revealed the largest declines among women aged 42.5–72.5 years (annual decreases of −5.5% to −7.2%). The longitudinal age curve demonstrated a peak incidence in middle-aged women (ages 40–50 years), followed by a progressive decline in older age groups. Cohort effects showed a pronounced reduction in risk across successive generations: women born after 1960 experienced markedly lower risks, with the 1993 birth cohort having less than half the risk compared to the 1958 reference cohort. Period effects further highlighted substantial declines after 2000, with the relative risk reduced to 0.67 in 2020 (Figure 5).

## 4. Discussion

In this population-based study in Taiwan spanning nearly three decades, we observed contrasting incidence trends for two major gynecologic cancers. From 1995 to 2022, cervical cancer incidence declined substantially, whereas corpus uteri cancer incidence increased steadily. These divergent trajectories are likely associated with differences in prevention strategies and evolving population-level risk factor profiles.

The sustained decline in cervical cancer incidence was most pronounced after 2000. When stratified by histological subtype, this reduction was primarily driven by a marked decrease in squamous cell carcinoma, whereas adenocarcinoma demonstrated a relatively stable trend over time. This pattern is consistent with prior evidence indicating that cytology-based screening programs are more effective in detecting and preventing squamous precancerous lesions than glandular lesions. Organized cytology-based screening, introduced in Taiwan in 1995, facilitates early detection and treatment of precancerous lesions and has been associated with reductions in invasive cervical cancer incidence [21,22]. Although HPV vaccination represents an important long-term preventive strategy, its population-level impact on invasive cervical cancer incidence is not expected to be observable within the current study period due to the long latency of cervical carcinogenesis. Therefore, the declining trends observed through 2022 may largely reflect the long-term effects of screening, with vaccination expected to contribute to additional reductions in future decades [23,24].

The sustained decline in cervical cancer incidence in Taiwan is likely associated with the long-standing Pap smear screening program. After organized screening was implemented in the mid-1990s, triennial screening participation reached approximately 50–53%, which was associated with substantial reductions in invasive cervical cancer incidence over time [22]. However, because our registry-based analysis lacks individual-level data on screening participation and HPV vaccination status, these associations should be interpreted cautiously rather than as direct causal effects.

In contrast, the increasing incidence of corpus uteri cancer likely reflects the combined influence of multiple population-level factors rather than a single causal determinant. The rising prevalence of obesity and metabolic disorders, delayed childbearing, declining parity, and prolonged lifetime estrogen exposure have all been associated with increased endometrial cancer risk in individual-level studies [25,26]. However, given the ecological nature of the present analysis, these factors should be interpreted as plausible contributors rather than direct causal explanations. The observed cohort effects, particularly the higher risks among women born after the late 1950s, may suggest cumulative generational exposure to these risk factors; however, causal inference cannot be established [27,28,29].

Our analyses further demonstrated that, although numerical differences in incidence trends were observed across geographic regions and urbanization levels, most confidence intervals overlapped, indicating an absence of statistically significant disparities. Notably, Joinpoint and age–period–cohort analyses suggested attenuation of cervical cancer decline and plateauing trends in certain subgroups, including eastern Taiwan and non-metropolitan areas. These patterns may be partially explained by differences in healthcare access and screening practices across regions. Prior population-based studies in Taiwan have reported lower Pap smear utilization among women in rural areas compared with those in urban settings, and reduced screening uptake has been associated with higher risks of cervical cancer detection [30]. However, because individual-level screening participation data were not available in the Taiwan Cancer Registry database used in this study, we were unable to directly evaluate geographic variation in screening adherence or attribute regional differences in incidence trends to disparities in screening participation. Similarly, the slowing but continued increase in corpus uteri cancer incidence across most regions underscores the widespread nature of underlying risk factors.

Age–period–cohort modeling provided additional insights into the temporal dynamics of these cancers. Corpus uteri cancer exhibited a significantly positive net drift, particularly among younger birth cohorts, consistent with the accumulation of metabolic and reproductive risk factors over time. Conversely, cervical cancer demonstrated a significantly negative net drift, with cohort effects indicating markedly reduced risks among women born after 1960. These cohort patterns are compatible with the potential long-term impact of screening-based prevention and broader generational shifts in exposure to risk factors [21,22].

From a clinical and public health perspective, these findings highlight two distinct priorities. For cervical cancer, maintaining high screening coverage and ensuring equitable access to screening services remain essential, particularly in regions showing signs of incidence plateau. Continued surveillance will be critical to evaluate the future population-level impact of HPV vaccination as vaccinated cohorts age into screening-eligible populations [23,24]. For corpus uteri cancer, the persistent rise in incidence emphasizes the need for heightened clinical awareness, risk stratification, and consideration of targeted prevention strategies for women with obesity or metabolic risk profiles [25,26]. At the health system level, these divergent trends underscore the importance of adaptive cancer control policies that address both successful prevention models and emerging disease burdens. Importantly, these interpretations are supported by the comprehensive coverage and high data quality of the Taiwan Cancer Registry, as reflected by its high completeness and the declining proportion of death-certificate-only cases over time.

### Limitations

This study has several limitations. First, our analysis was based on the Taiwan Cancer Registry, which has improved in completeness and diagnostic accuracy in recent years, but earlier data may still have been subject to underreporting or misclassification. Second, individual-level risk factor data (e.g., obesity, metabolic syndrome, contraceptive use, hormone therapy, HPV infection status, lifestyle factors) and screening participation information were unavailable, preventing direct evaluation of underlying causes and geographic differences in screening adherence. Third, histological subtypes of endometrial carcinoma were not consistently reported across all study years. Specifically, histologic detail sufficient to classify endometrial carcinoma into type I (endometrioid) and type II (serous/clear cell) categories was consistently available only after 2018. Consequently, subtype-specific trend analyses could not be performed across the full study period. Fourth, urbanization level was defined using administrative categories reported in the Taiwan Cancer Registry annual reports, which may not fully capture within-city heterogeneity or finer township- or district-level urbanization characteristics. Finally, interpretation of age–period–cohort results should be cautious due to the inherent identifiability problem of APC models, and the observed cohort and period effects should be interpreted as descriptive temporal patterns rather than causal relationships.

## 5. Conclusions

In conclusion, this population-based study demonstrates markedly divergent long-term incidence trends for two major gynecologic cancers in Taiwan from 1995 to 2022. Cervical cancer incidence declined substantially over the study period, patterns that are compatible with the long-term impact of organized cytology-based screening programs, with cohort and period effects suggesting durable population-level benefits. In contrast, the incidence of corpus uteri cancer increased steadily across age groups, birth cohorts, geographic regions, and urbanization levels, underscoring the growing burden potentially associated with metabolic, reproductive, and lifestyle-related risk factors in contemporary Taiwanese society.

These findings highlight the dual challenges facing gynecologic cancer control. Continued efforts are required to maintain high screening coverage and equitable access for cervical cancer prevention, particularly in subgroups showing signs of incidence plateau. At the same time, the rising burden of corpus uteri cancer calls for heightened clinical awareness, improved risk stratification, and the development of targeted prevention strategies addressing obesity and metabolic health. Ongoing surveillance using high-quality registry data will be essential to monitor future trends and to guide adaptive public health policies aimed at reducing gynecologic cancer burden in Taiwan.

## Figures and Tables

**Table 1:** Annual percent change (APC) and average annual percent change (AAPC) in age-standardized incidence rates (ASRs) of corpus uteri cancer and cervical cancer by geographic region, Taiwan, 1995–2022.

Cancer Type	Region	Period	APC (95% CI)	*p*-Value	AAPC (95% CI)	*p*-Value
Corpus uteri	Northern Taiwan	1995–2022	5.81 (5.39, 6.23)	<0.001 *	5.81 (5.39, 6.23)	<0.001 *
	Central Taiwan	1995–1997	23.66 (4.11, 46.88)	0.018 *	7.24 (5.77, 8.72)	<0.001 *
		1997–2015	7.41 (6.76, 8.06)	<0.001 *		
		2015–2022	2.54 (0.20, 4.92)	0.034 *		
	Southern Taiwan	1995–2009	9.06 (8.02, 10.11)	<0.001 *	7.03 (6.61, 7.76)	<0.001 *
		2009–2022	4.89 (3.77, 6.02)	<0.001 *		
	Eastern Taiwan	1995–2000	24.09 (8.91, 41.38)	0.002 *	8.55 (5.84, 11.33)	<0.001 *
		2000–2022	5.30 (3.85, 6.77)	<0.001 *		
Cervical cancer	Northern Taiwan	1995–2000	−1.72 (−4.82, 1.49)	0.271	−4.75 (−6.35, −3.11)	<0.001 *
		2000–2003	−14.46 (−25.9, −1.25)	0.035 *		
		2003–2016	−5.06 (−5.86, −4.25)	<0.001 *		
		2016–2022	−1.37 (−3.74, 1.05)	0.246		
	Central Taiwan	1995–1998	8.67 (−6.01, 25.66)	0.246	−3.93 (−5.70, −2.12)	<0.001 *
		1998–2012	−7.41 (−8.81, −5.99)	<0.001 *		
		2012–2022	−2.51 (−4.69, −0.28)	0.030 *		
	Southern Taiwan	1995–1997	12.88 (−4.74, 33.75)	0.152	−3.90 (−5.17, −2.61)	<0.001 *
		1997–2011	−6.79 (−7.62, −5.96)	<0.001 *		
		2011–2022	−2.97 (−4.07, −1.85)	<0.001 *		
	Eastern Taiwan	1995–2013	−6.95 (−8.64, −5.22)	<0.001 *	−4.78 (−6.68, −2.84)	<0.001 *
		2013–2022	−0.30 (−5.37, 5.04)	0.907		

Abbreviations: APC, annual percent change; AAPC, average annual percent change; ASR, age-standardized incidence rate; CI, confidence interval. * *p* < 0.05.

**Table 2:** Annual percent change (APC) and average annual percent change (AAPC) in age-standardized incidence rates (ASRs) of corpus uteri cancer and cervical cancer by urbanization level, Taiwan, 1995–2022.

Cancer Type	Urbanization Level	Period	APC (95% CI)	*p*-Value	AAPC (95% CI)	*p*-Value
Corpus uteri	Metropolises	1995–1997	15.91 (0.61, 33.52)	0.042 *	6.37 (5.18, 7.57)	<0.001 *
		1997–2014	6.42 (5.84, 7.00)	<0.001 *		
		2014–2022	4.01 (2.42, 5.63)	<0.001 *		
	Non-metropolises	1995–2008	10.04 (9.00, 11.08)	<0.001 *	6.99 (6.35, 7.63)	<0.001 *
		2008–2022	4.24 (3.36, 5.12)	<0.001 *		
Cervical cancer	Metropolises	1995–1998	6.05 (−1.31, 13.96)	0.104	−4.48 (−5.40, −3.54)	<0.001 *
		1998–2006	−9.57 (−11.29, −7.82)	<0.001 *		
		2006–2022	−3.73 (−4.26, −3.19)	<0.001 *		
	Non-metropolises	1995–2017	−5.96 (−6.55, −5.37)	<0.001 *	−4.90 (−5.98, −3.81)	<0.001 *
		2017–2022	−0.10 (−5.83, 5.98)	0.972		

Abbreviations: APC, annual percent change; AAPC, average annual percent change; ASR, age-standardized incidence rate; CI, confidence interval. * *p* < 0.05.

## Data Availability

The original contributions presented in this study are included in the article. Further inquiries can be directed to the corresponding authors.

## Abbreviations

The following abbreviations are used in this manuscript:

ASR	Age-standardized incidence rate
HPV	Human papillomavirus
APC	Annual percent change
AAPC	Average annual percent change
